# Supplementary material for: High Glucose Reduces the Paracellular Permeability of the Submandibular Gland Epithelium via the MiR-22-3p/Sp1/Claudin Pathway
Source: Cells. 2021 Nov 19;10(11):3230. doi: 10.3390/cells10113230 (PMC8617860; doi:10.3390/cells10113230)
Supplement: Supplementary file 1 [file cells-10-03230-s001.zip › cells-1415369-supplementary.pdf]

Table S1. Information of the patients

Patient	Gender	Age (years)	Disease
P1	Female	46	Mandible tumor
P2	Male	69	Tongue SCC
P3	Male	62	Buccal SCC
P4	Female	67	Gingiva SCC
P5	Male	75	Gingiva SCC
P6	Male	38	Tongue SCC
P7	Male	74	Buccal SCC
P8	Male	68	Mandible tumor
P9	Male	67	Gingiva SCC
P10	Female	43	Tongue SCC

SCC, squamous cell carcinoma.

Table S2. Information of primary antibodies

Antibodies	Catalog	Source	Dilution
Anti-claudin-1	BS1063	Bioworld Technology	1:1,000 (WB), 1:100 (IHC)
Anti-claudin-3	BS1067	Bioworld Technology	1:1,000 (WB), 1:100 (IHC)
Anti-claudin-4	BS1068	Bioworld Technology	1:1,000 (WB), 1:100 (IF)
Anti-claudin-7	BS1070	Bioworld Technology	1:1,000 (WB)
Anti-claudin-10	38-8400	Thermo Fisher Scientific	1:1,000 (WB)
Anti-Sp1	ab13370	Abcam	1:200 (ChIP)
Anti-Sp1	NB600-233	NOVUS Biologicals	1:10,000 (WB)
Anti-β-actin	#4970	Cell Signaling Technology	1:1,000 (WB)

Sp1, specificity protein-1; WB, western blot; IHC, immunohistochemistry; IF, immunofluorescence; ChIP, chromatin immunoprecipitation.

Table S3. Primers sequences used in RT-PCR

Gene	Forward primer (5'-3')	Reverse primer (5'-3')
Mouse		
Claudin-1	GACAGGAGCAGGAAAGTAGGA	CTTTGGAATTAGGCAGAACGA
Claudin-2	CCATGGCCTCCCTTGGCGTCC	CACACATACCCAGTCAGGCTG
Claudin-3	TTTCTTTGTCCATTCGGCTTG	ACCGTACCGTCACCACTACCA
Claudin-4	GATCTTGGCCTTGACGGTCTC	CTCTGGATGAACTGCGTGGTG
Claudin-5	CCATGGGGTCTGCAGCGTTGG	GGCGAACCAGCAGAGCGGCAC
Claudin-7	CCATGGCCAACTCGGGCCTGCAAC	TCACACGTATTCCTTGGAGG
Claudin-10	CCATGGGTAGCACGGCCTTGG	TTAGACATAGGCATTTTTATC
Sp1	TCCACCTGCTGTCTCATC	TACCACCCTAACACCCAT
β-actin	CCAACCGTGAAAAGATGACC	CCAGAGGCATACAGGGACAG
Human		
Claudin-1	GCAGAAGATGAGGATGGCTGT	CCTTGGTGTTGGGTAAGAGGT
Claudin-3	GGACTTCTACAACCCCGTGGT	AGACGTAGTCCTTGCGGTCGT
Claudin-4	CAAGGCCAAGACCATGATCGT	GCGGAGTAAGGCTTGTCTGTG
Claudin-7	CTCGAGCCCTAATGGTGGTCT	CCCAGGACAGGAACAGGAGAG
Sp1	AGACAGTGAAGGAAGGGGCT	GCGTTTCCCACAGTATGACC
β-actin	CTACCTCATGAAGATCCTCACCGA	TTCTCCTTAATGTCACGCACGATT
Rat		
Claudin-1	CATCATCTTCTAAGCACCTCA	GGGACAACATCGTGACTG
Claudin-3	TCTTGGCCTTGGCCGTCT	TCTATCCTACTGGCAGCCTTCG
Sp1	GGACAGTTGAGCAGCATT	CCATCATCATTCGGACAC
β-actin	CCCATCTATGAGGGTTACGC	TTTAATGTCACGCACGATTTC

Sp1, specificity protein-1.

Table S4. Sequences of claudin-1 and -3 shRNA

Gene	Sequence (5’-3’)
Claudin-1 shRNA	ACCAGAGCCTTGATGGTAATTGGCATCCT
Claudin-3 shRNA	CTCTCATCGTGGTGTCTATCCTACTGGCA

Table S5. Sequences of Sp1 siRNA

Gene	Forward sequence (5'-3')	Reverse sequence (5'-3')
rno-Sp1-siRNA	GCAAGUUCUGACAGGUCUATT	UAGACCUGUCAGAACUUGCTT
NC siRNA	UUCUCCGAACGUGUCACGUdTdT	ACGUGACACGUUCGGAGAAdTdT
PC siRNA	CCCUCACAAGAGGAUUGAAdTdT	UUCAAUCCUCUUGUGAGGGdTdT

Sp1, specificity protein-1; NC, negative control; PC, positive control.

Table S6. Primers sequences of claudin-1 promoter fragments

Target sequence (bp)	Sequence		Amplicon (bp)
	Forward primer (5'-3')	Reverse primer (5'-3')	
-400 to -283	CTAGACAGGATTAGGCTGTCGAGG	GTTGTGCCTTAGGACCCATTTCT	201
-284 to -84	ATTTGGCCACGAAACCACCA	AGCCAGGAGGTTAGCGCTGA	200
-87 to 110	CTGAGACTCCATCACCTTCGG	CCAGCATAGGAGTAAATCTTCC	197
111 to 315	GGACAACATCGTGACTGCTCAG	GCTACTACAGAGGACAAGGGCT	204

Table S7. Primers sequences of claudin-3 promoter fragments

Target sequence (bp)	Sequence		Amplicon (bp)
	Forward primer (5'-3')	Reverse primer (5'-3')	
-1677 to -1480	TCGATGAGGTGCCTACTGACAT	CTTGGGGTCCTTTCTTCACCAG	197
-739 to -562	GGTGCTACATTTCCATCCACC	TGAAGTGCAAATCACCAATTG	177
-364 to -146	GTGAAGAGGGTTGGGGAGTAC	CACGCTAACGGCTCCTTTCC	218
-147 to 52	CTCCGTCCATTGCCATCC	CACACGATGGTGCACAGC	199
53 to 199	TGTGCACCATCGTGTGCT	CAGCGAGTCGTACATCTTGC	146
200 to 396	ACTCGCTGCTGGCCCTGC	TAAGGTGAGCACAGCCGC	196

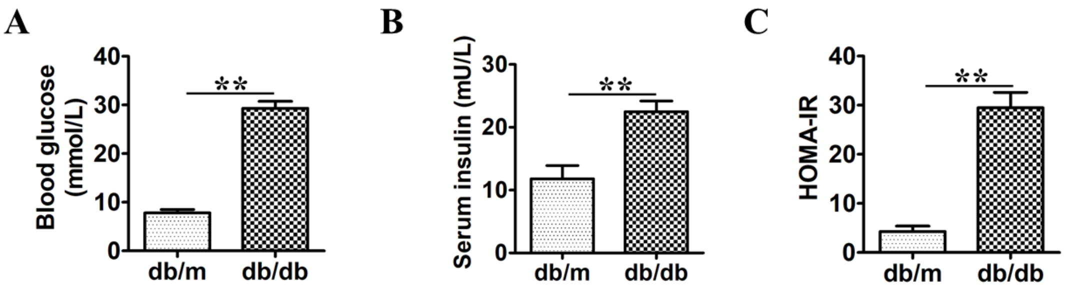

Figure S1. Confirmation of diabetes in db/db mice. (**A**) Blood glucose levels of db/m and db/db mice. (**B**) Serum insulin levels of db/m and db/db mice. (**C**) Insulin resistance index (HOMA-IR) of db/m and db/db mice. n = 5, **$p < 0.01$.

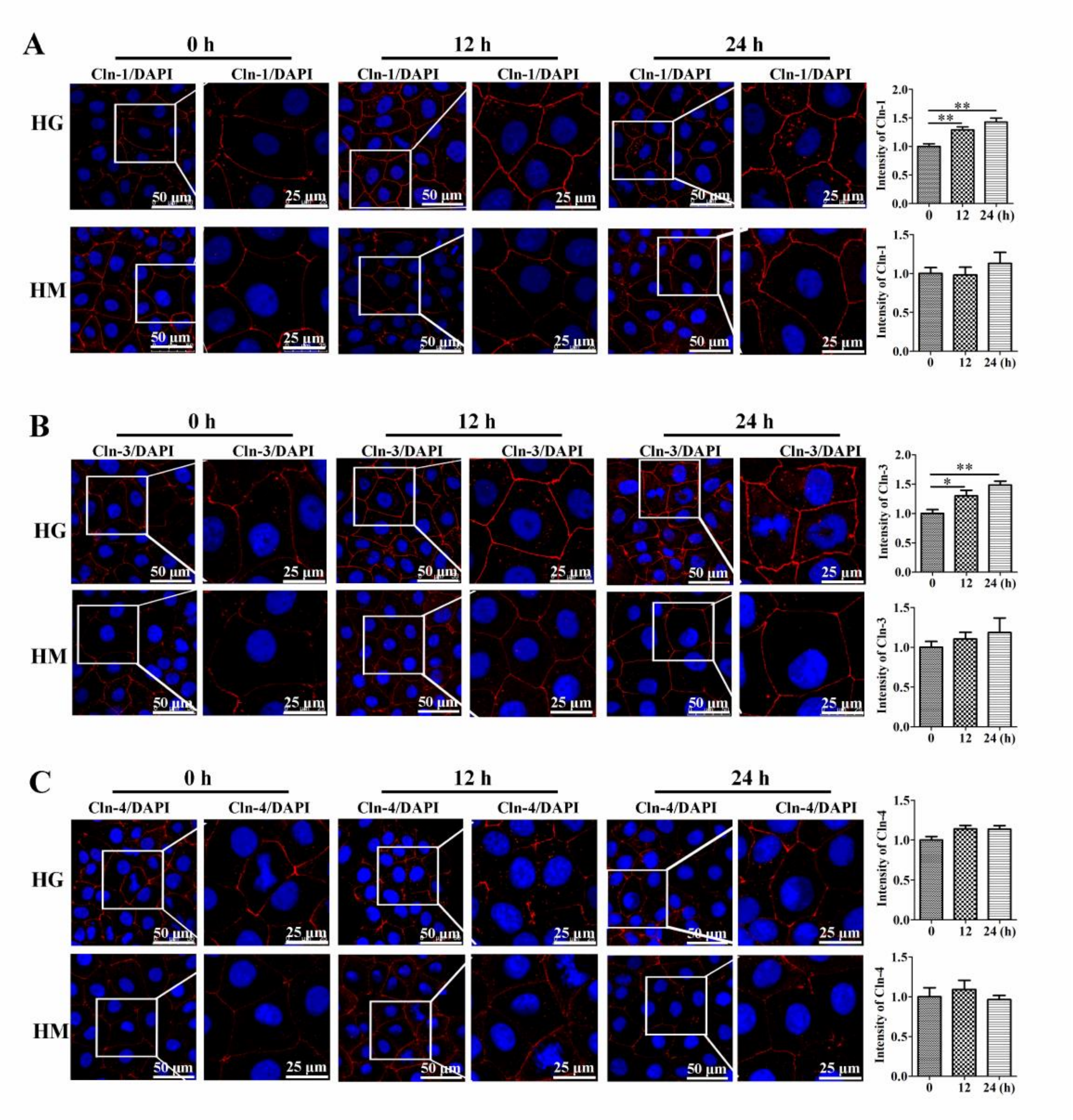

Figure S2. Immunofluorescence staining of claudin-1(Cln-1) (A), Cln-3 (B) and Cln-4 (C) in high glucose (HG) and high mannitol (HM)-treated SMG-C6 cells.

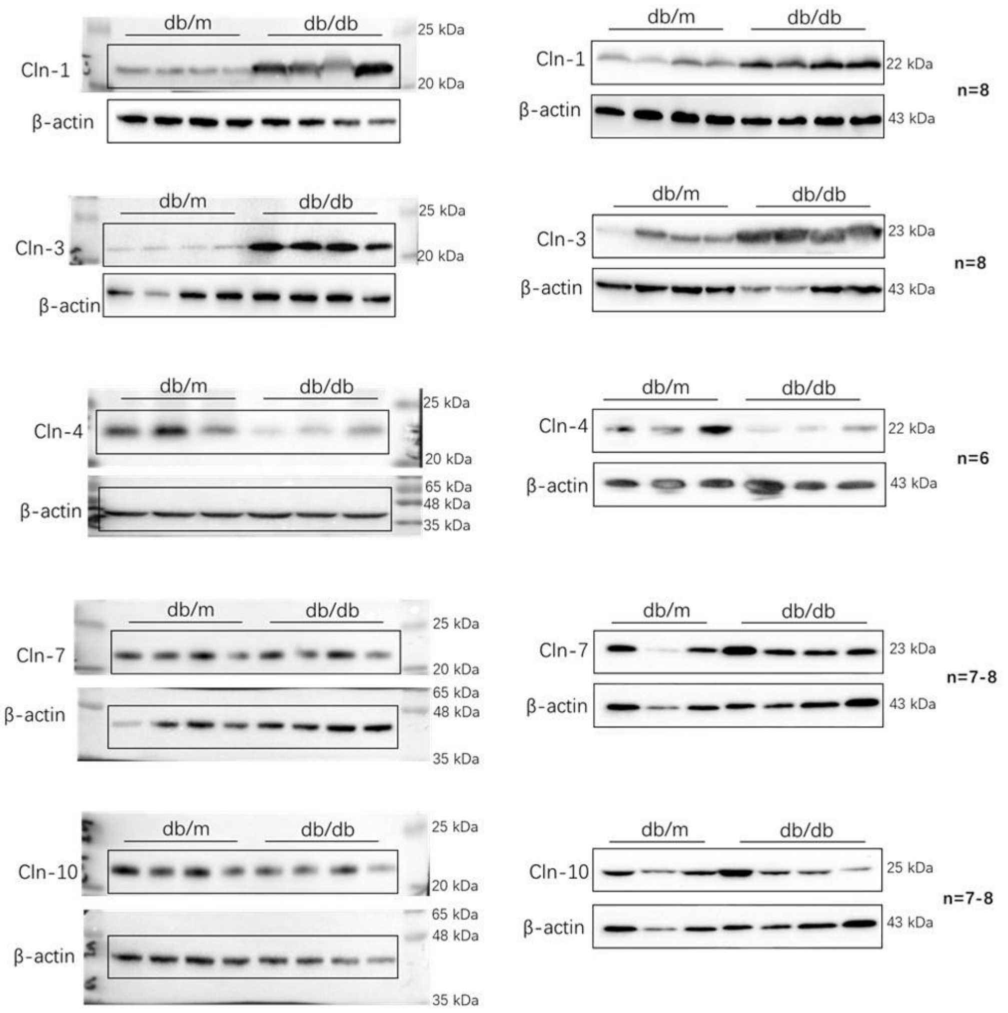

Figure S3. All western blot bands of claudin-1 (Cln-1), Cln-3, Cln-4, Cln-7, and Cln-10 in mouse SMGs.

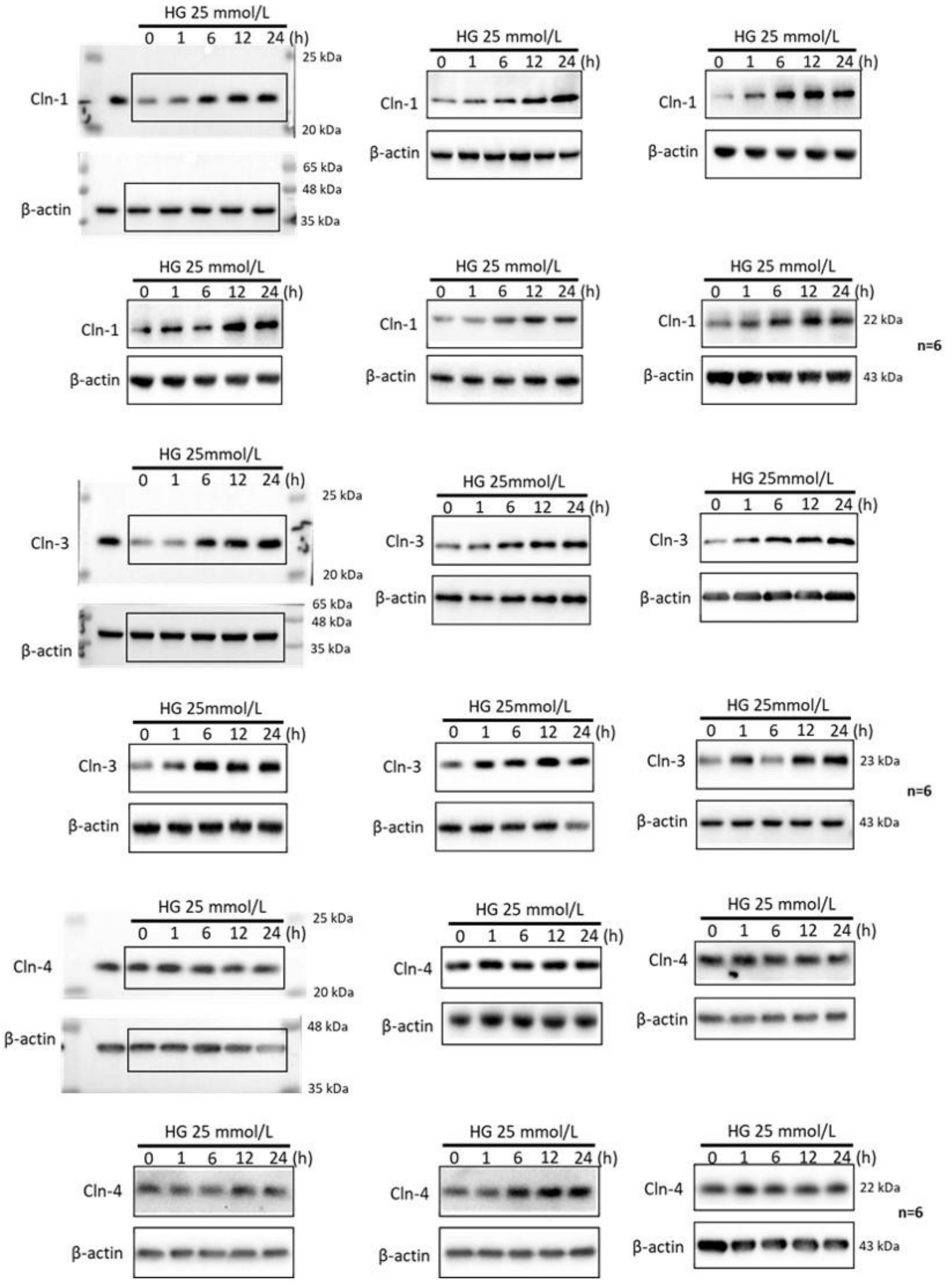

Figure S4. All western blot bands of claudin-1 (Cln-1), Cln-3, and Cln-4 in high glucose (HG = 25 mmol/L)-treated SMG-C6 cells.

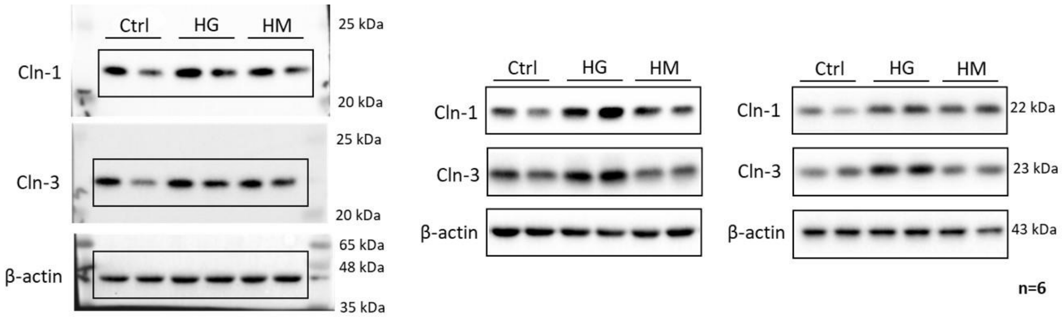

Figure S5. All western blot bands of claudin-1 (Cln-1) and Cln-3 in the control (Ctrl), high glucose (HG = 25 mmol/L), and high mannitol (HM = 25 mmol/L) groups.

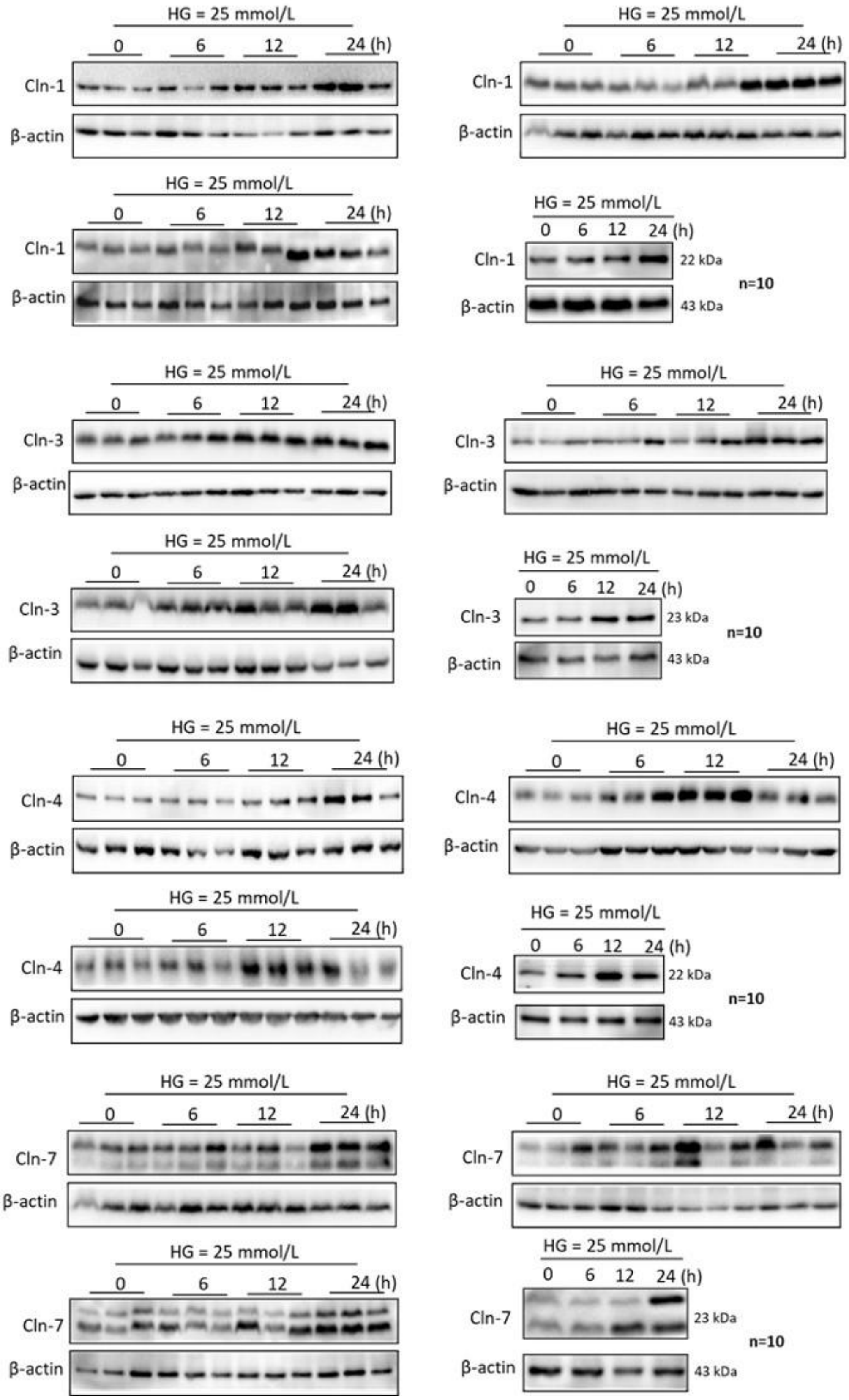

Figure S6. All western blot bands of claudin-1 (Cln-1), Cln-3, Cln-4, and Cln-7 in high glucose (HG = 25 mmol/L)-treated human SMGs.

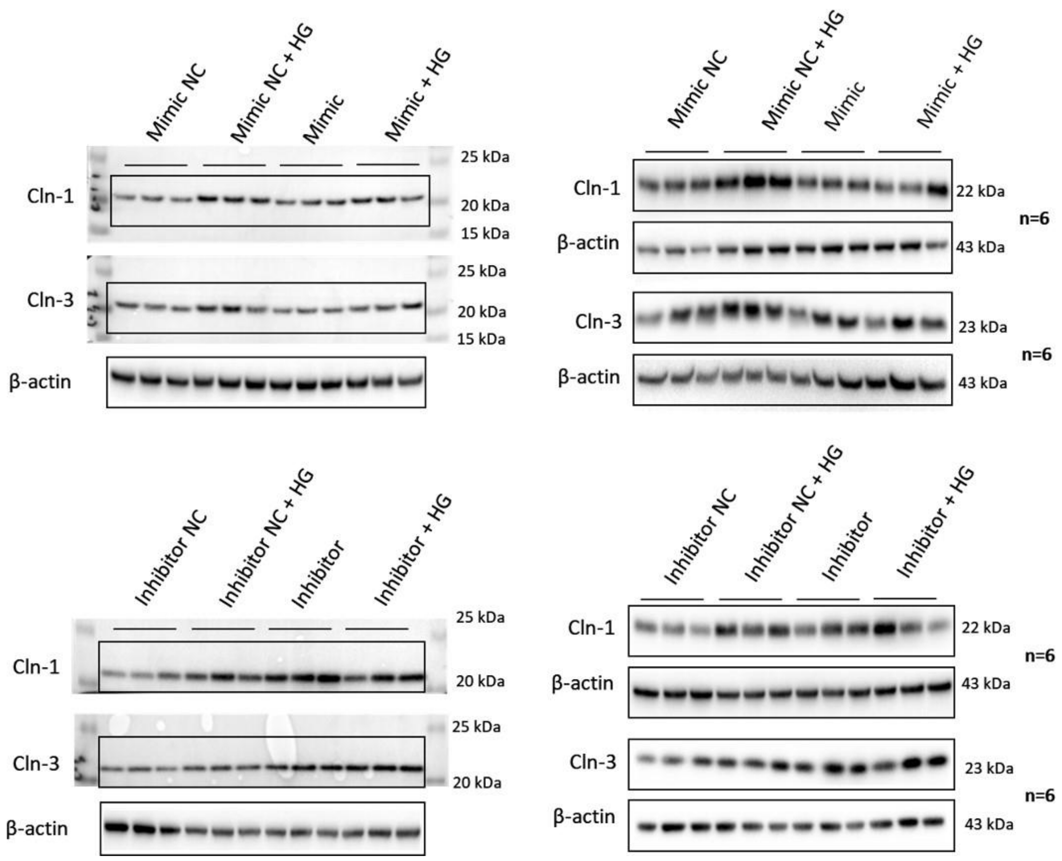

Figure S7. All western blot bands of Cln-1 and Cln-3 after transfecting SMG-C6 cells with the miR-22-3p mimic or inhibitor. NC, negative control; HG, high glucose.

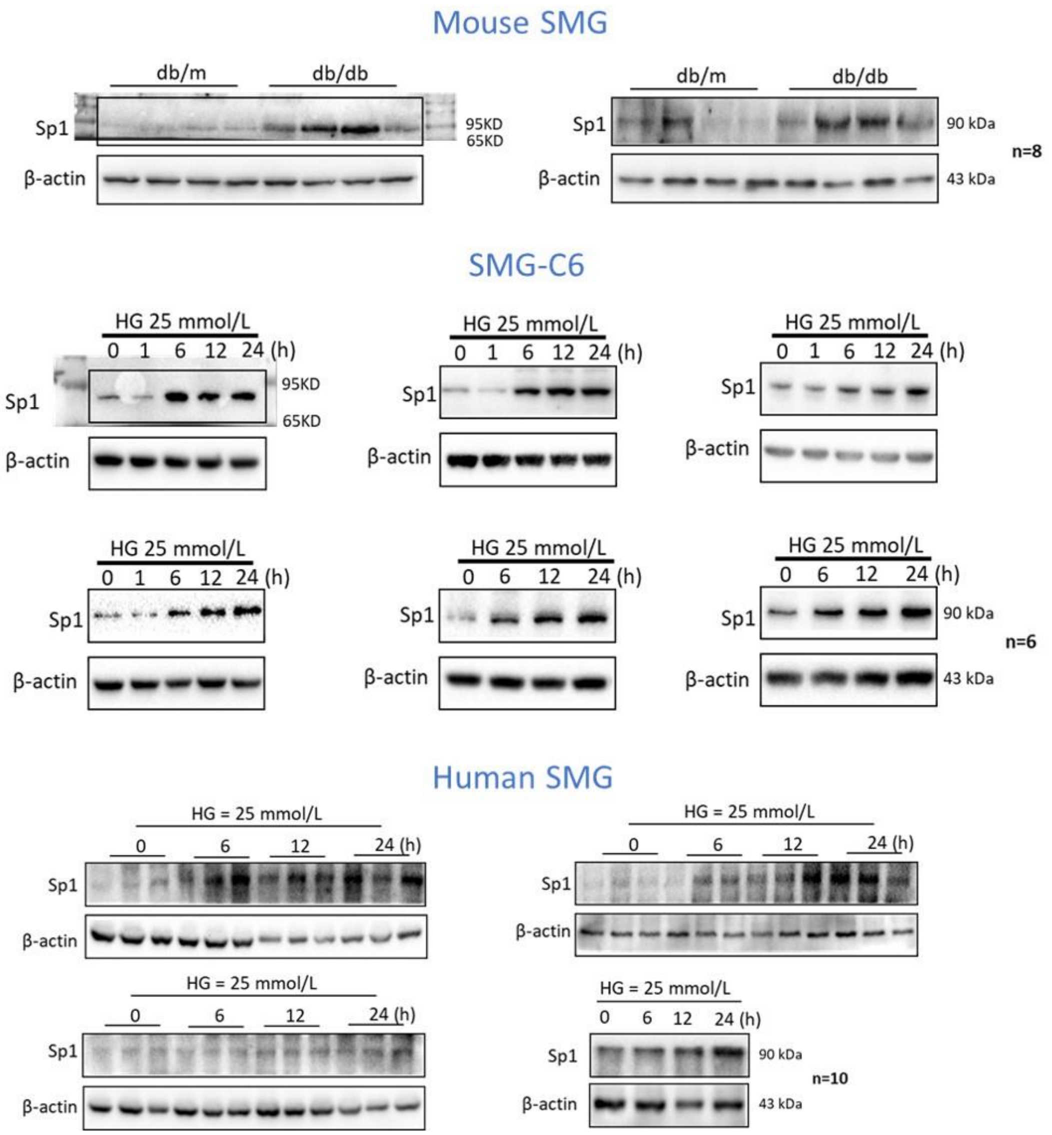

Figure S8. All western blot bands of Sp1 in mouse SMGs, high glucose (HG = 25 mmol/L)-treated SMG-C6 cells, and HG-treated human SMGs.

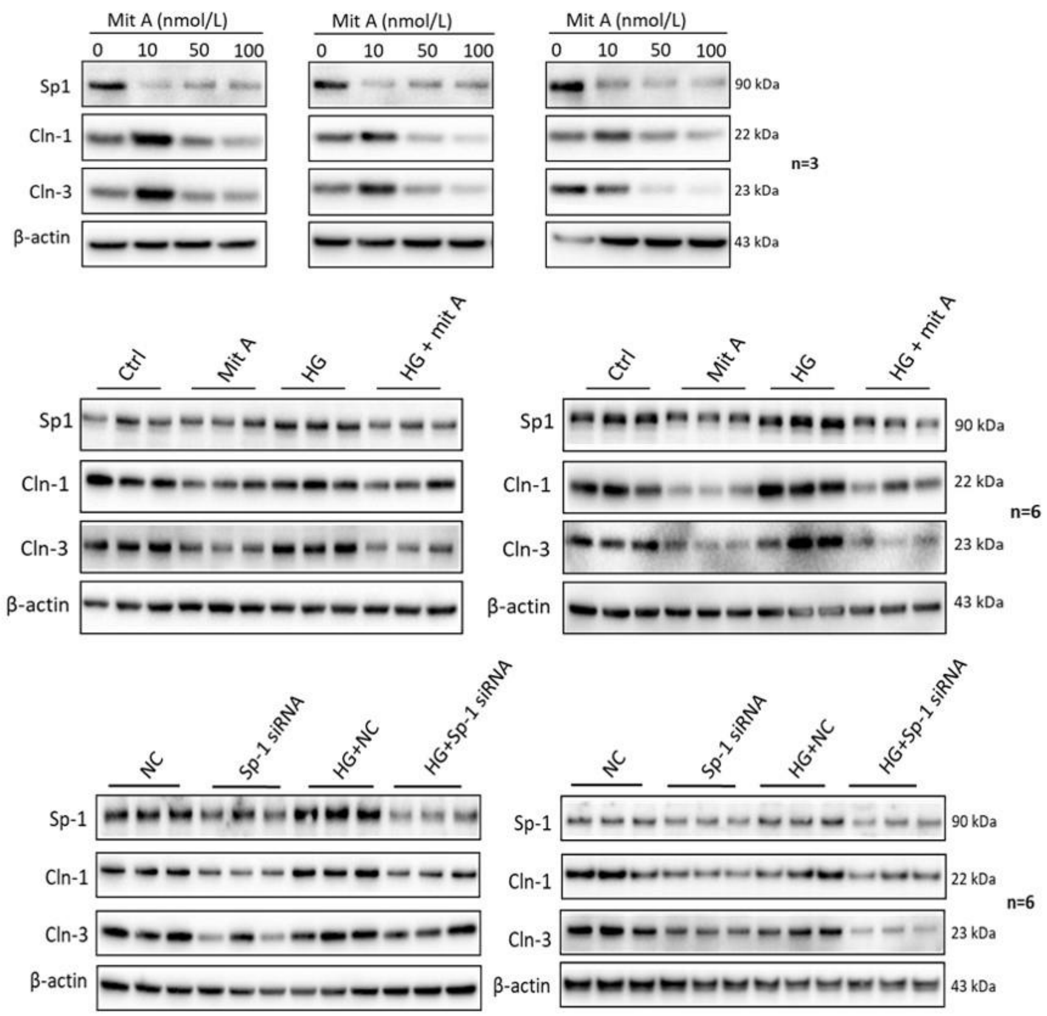

Figure S9. All western blot bands of Sp1, Cln-1 and Cln-3 after incubating SMG-C6 cells with Sp1 inhibitor mithramycin A (mitA) or transfecting SMG-C6 cells with Sp1-specific siRNA. Ctrl, control; NC, negative control; HG, high glucose.

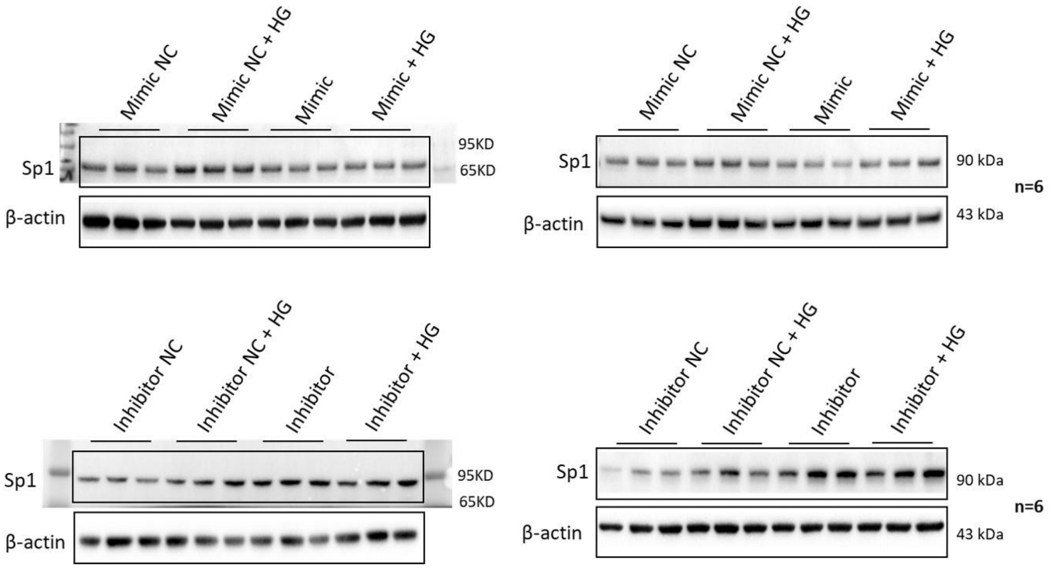

Figure S10. All western blot bands of Sp1 after transfecting SMG-C6 cells with the miR-22-3p mimic or inhibitor.
